# Selection of best videos of the year for 2021

**DOI:** 10.1590/S1677-5538.IBJU.2022.01.03

**Published:** 2021-12-21

**Affiliations:** 1 Department of GU Oncology Moffitt Cancer Center FL USA Department of GU Oncology Moffitt Cancer Center, FL, USA; 2 Department of Tumor Biology Moffitt Cancer Center FL USA Department of Tumor Biology Moffitt Cancer Center, FL, USA

## COMMENT

It is that special time of the year where we celebrate the extraordinary surgical achievements and commitment to innovation of our contributors to the video section of the **International Brazilian Journal of Urology**. This past year has seen ongoing incredible challenges to our global healthcare community in caring for patients impacted by COVID-19 during this ongoing pandemic. In consequence, many parts of the world have had to postpone elective surgeries for at times extended periods and also this has placed an incredible constraint on available surgical resources in caring for our surgical patients. Despite that, I am proud to say that we have continued to see an unwavering commitment by urologists across the world in offering first class surgical care to patients and there is no better example of this as depicted in this year's selection of the best video submissions and publications within our journal which are selected on a host of factors including originality, quality of the video and narration, and potential impact on improving surgical outcomes for patients through its widespread adoption by colleagues. In this regard, I am proud to announce that the 1st Prize for best video of the year is given to Dr. Tobias-Machado et al. from São Paulo, Brazil in the video entitled “**Robotic parastomal hernia repair**” published in March-April 2021 (1). In this video submission, the authors beautifully depict how a truly minimally invasive robotic platform can be used to repair such parastomal defects with truly excellent surgical outcomes (minimal blood loss, discharge within 24 hours, and very acceptable surgical duration of close to 2 hours in both cases). As we all know, the management of parastomal hernias can be quite challenging and often disappointing in terms of outcomes and prolonged recovery/convalescence, with this technique clearly having significant merits in minimizing these sequelae. The second prize is awarded to the video entitled “**Robot-assisted simple prostatectomy: the evolution of a surgical technique**” by Dr. Rodrigues et al. from São Paulo, Brazil and published in May-June 2021 (2). This video submission is quite elegant and illustrative with these surgeons from the group of Dr. Rafael Coelho illustrating 3 distinct and innovative surgical approaches (1-transversal incision of the bladder neck, 2- transvesical approach, and 3- Retzius sparing) to robotic assisted simple prostatectomy. This submission very nicely depicts how any of these 3 surgical approaches can be completed with excellent outcomes and offering a host of surgical options that colleagues can chose from or consider in adding to their surgical armamentarium. The 3rd Prize for best video of the year is awarded to Dr. Ahluwalia et al. from Mumbai, India in their video entitled “Robotic radical nephrectomy and level II inferior vena cava thrombectomy: exploring the new frontiers” and published in January-February 2021 (3). In this video, the authors nicely depict how minimally invasive robotic surgical techniques can be used to manage such highly complex surgical cases as renal tumors with IVC tumor thrombi. Strict adherence to surgical vascular principles as meticulous proximal/distal IVC control and of the contralateral renal vein are critical to minimize significant intraoperative blood loss and adverse surgical outcomes. There is no question some level of selection is critical in determining which renal tumors and IVC tumor thrombi can and should be explored using a minimally invasive robotic technique versus a more conventional open technique including proximal level and volume of the IVC thrombi, suspicion of caval wall invasion on preoperative imaging (preferably MRI with intravenous contrast) requiring possible vascular patching or sleeve grafting, and most importantly surgeon and surgical team's skillset and patients underlying co-morbidities (ie cardiac, pulmonary).

In conclusion, I wanted to thank each and every one of you for supporting our journal through truly unprecedented challenging times which has tested all of us in terms of our resilience and ability to adapt. I very much send my very best wishes to all of you and your families to remain safe and in good spirits. Very much looking forward to seeing you all in person within the coming months and years.

Warmest regards and best wishes,
